# Functional outcomes after combined iris and intraocular lens implantation in various iris and lens defects

**DOI:** 10.1186/s12886-020-01621-8

**Published:** 2020-09-15

**Authors:** Christian S. Mayer, Andrea M. Hoffmann, Phillipp Prahs, Lukas Reznicek, Ramin Khoramnia

**Affiliations:** 1 Department of Ophthalmology, University Hospital Heidelberg, University of Heidelberg, Im Neuenheimer Feld 400, 69120 Heidelberg, Germany; 2 Department of Ophthalmology, University Hospital Klinikum rechts der Isar, Technical University of Munich, Munich, Germany; 3 Department of Ophthalmology, University of Regensburg, Regensburg, Germany

**Keywords:** Aniridia, Pupillary reconstruction, Ocular trauma, Eye injury, IOL

## Abstract

**Background:**

To assess the functional outcomes after combined iris and intraocular lens (IOL) repair in aniridia patients.

**Methods:**

Retrospective observational study in 59 aniridic and aphakic eyes for ArtificialIris (AI) and IOL reconstruction. The iris prostheses were placed together with the IOL in the capsular bag using an injection system or were fixed by transscleral suturing of the IOL and AI. The primary outcomes measured were visual acuity, contrast and glare sensitivity (Pelli-Robson chart for photopic and dark adaptometer for mesopic conditions), intraocular pressure, endothelial cell density (ECD) and patient impairment.

**Results:**

Blunt trauma (37 eyes) and penetrating injuries (16 eyes) were observed more frequently than congenital aniridia (1 eye), iatrogenic causes (1 eye), aniridic state after severe iritis (2 eyes) or iris tumor (2 eyes). Monocular CDVA improved significantly (*p* < 0.0001) from median 0.7 logMAR (0.0–1.98) to 0.3 logMAR (− 0.08–2.0). Median pupillary area could significantly (*p* < 0.0001) be reduced by 79.3% from 51.27 mm^2^ (17.91 to 98.23) to 8.81 mm^2^ (4.16 to 8.84). Median ECD decreased from 2646.0 mm^2^ to 2497.5 mm^2^ (*p* = 0.007). Contrast and glare sensitivity improved significantly (*p* = 0.008) in photopic light conditions from 0.9 (0.0–1.95) to 1.35 (0.0–1.8). Patients reported to be highly satisfied with the functional improvement.

**Conclusion:**

The flexible ArtificialIris seems to be a safe and effective iris prosthesis in combination with an IOL having functionally and cosmetically exceptional reconstruction options.

## Background

Iris defects either from congenital, traumatic or other causes come in various dimensions and may lead to considerable visual impairment. Photophobia, increased glare from bright light, decreased visual acuity and contrast sensitivity are the most common symptoms in aniridic patients. These patients may also suffer extensively from the unaesthetic appearance of their eyes, particularly in case of substantial iris loss.

The management of congenital aniridia can be very challenging because of the complexity of the disease and the association with severe ocular comorbidities [1]. More often, iris abnormalities result from traumatic injury, e.g. blunt trauma or penetrating eye injuries. Posttraumatic eyes may also present morphological and functional alterations due to ocular injuries, for example corneal damage and lens lesion [2]. In respect of the high rate of secondary impairment, careful management is needed to improve the patients’ quality of vision and cosmetic appearance.

Conservative therapeutic approaches for aniridia include iris print contact lenses, corneal tattooing, sunglasses, occlusion intraocular lenses and simple iris suturing for small defects of approximately 2 clock hours. However, in case of large iris defects, implantation of a prosthetic iris device may be more appropriate.

Currently, several models are available, including the foldable custom-made CUSTOM*FLEX*® ARTIFICIAL*IRIS* from HumanOptics (Erlangen, Germany). The device received the CE marking in 2011 and is the only iris prosthesis that received FDA approval (2018) for the treatment of vision and cosmetic problems arising from congenital, surgical or traumatic aniridia in adults and children.

It is available with or without embedded polymer fiber meshwork and can be implanted through small corneal incisions within the capsular bag or ciliary sulcus with or without scleral fixation. The various implantation techniques of the artificial iris for the management of complete or partial aniridia have been extensively described elsewhere [3]. The favorable outcomes in terms of functional vision, aesthetics and patient satisfaction have also been largely reported in the literature [4–9]. Nonetheless, complications may occur, but as shown in our previously published work, the complication rate decreases indirectly proportional to the increasing experience and the learning curve of the surgeon [10].

In this retrospective study, we focused on the functional rehabilitation after implantation of the artificial iris in 59 eyes with large aniridic defects and simultaneous need for intraocular lens (IOL) implantation due to cataract or aphakia. We therefore evaluated the subjective patients’ impairment in terms of glare, contrast sensitivity under photopic and mesopic conditions and the subjective impairment with a questionnaire pre- and postoperatively.

## Methods

This retrospective observational case series comprised 59 eyes of 56 patients who were implanted with the CUSTOM*FLEX*® ARTIFICIAL*IRIS* and an intraocular lens in one session over a period of 7 years (2011/09–2018/04). Patients were referred to the clinic for ocular trauma in 53 eyes (89.83%), iris tumor in 2 eyes (3.39%), aniridic state after severe iritis in 2 eyes (3.39%), congenital aniridia in one eye (1.69%) and iatrogenic aniridia in one eye (1.69%).

Prior to surgery, details of the surgical procedure were explained to all patients and written and verbal informed consent was obtained from all participants. The study was conducted according to the tenets of the Declaration of Helsinki, and approval by the Institutional Review Board was obtained.

To evaluate the severity of ocular injuries, we initially classified the eyes based on the Ocular Trauma Score (OTS) according to Kuhn et al. [11]. The OTS evaluating system is calculated by assigning numerical raw points to six variables: initial visual acuity, rupture of the globe, penetrating injury, endophthalmitis, retinal detachment, and afferent pupillary defect. The score is then stratified into five categories (1 to 5): the higher the score the less the traumatic level.

### Surgical management

**Fig. 1:**
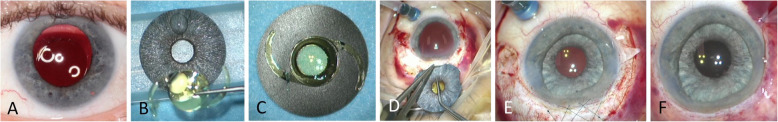
From preoperative state (**a**), combined preparation of the AI and IOL (**b** and **c**), implantation of the “sandwich” through a sclerocorneal incision (**c**) and suturing to the sclera (**e**) to postoperative outcome (**f**)

All surgeries were performed by one surgeon (CM) in general anesthesia. The preparation of the artificial iris and the different implantation procedures have been previously described in detail [3]. In brief, before implantation of the artificial iris, phakic patients underwent either standard cataract surgery with IOL implantation in the capsular bag or an IOL was sutured to the sclera in cases where no capsular bag was available. Patients received either a one-piece hydrophilic acrylic IOL (ASPIRA-aAY, HumanOptics AG, Erlangen, Germany) in the capsular bag (*n* = 15) respectively sutured to the AI (*n* = 30) (Fig. 1) or a PMMA IOL for scleral fixation (*n* = 14) (Type 81B, Morcher GmbH, Stuttgart, Germany) when they had inadequate capsule or zonula support or were primarily aphakic.

Artificial iris implants were placed through an approx. 7.0 mm superior sclerocorneal incision into the ciliary sulcus and then sutured. In 15 eyes, however, the IOL and artificial iris were placed in the capsular bag. For a more detailed description we can refer to a previous publication [3]. We generally choose the artificial iris version with embedded fiber in cases of suturing the iris prosthesis; otherwise the fiber free version was implanted. If suturing was not necessary, the prosthesis was injected through a 2.8 mm-sized superior corneal incision using a shooter system (Viscoject 2.2, Medicel AG, Thal, Switzerland). Surgeries lasted between 20 and 125 min (median 74.0 min; 71.69 ± 31.14 min).

### Study parameters

All patients were examined preoperatively and followed-up at least 3 months after surgery. A comprehensive ophthalmic examination including biomicroscopic evaluation of the anterior and posterior eye segment, Goldmann applanation tonometry and the measurement of intraocular pressure (IOP) were performed at all visits.

Study parameters assessed preoperatively and at the 5 months follow-up were the following: corrected distance visual acuity (CDVA); photopic and mesopic contrast sensitivity (CS); endothelial cell density (ECD), pupil area and patient’s satisfaction in terms of changes in symptoms of light sensitivity and daytime glare results.

All visual tests were performed monocularly. Distance visual acuity was measured using standardized optotypes at 5 m distance. Contrast sensitivity under photopic and mesopic conditions was measured with best correction using the Pelli-Robson chart (test distance of 1 m). Furthermore, an evaluation of the sensitivity to glare was performed with the Nyktometer 500 (test disc 505, Rodenstock GmbH, Munich, Germany). The Nyktometer test disc 505 consists of 12 Landolt rings with four different contrast levels. Time for pre-adaptation to mesopic light conditions was at least 3 min for each patient. Both tests were performed with glare (Pelli-Robson chart; Nyktometer 0.3 cd/m^2^).

The ECD, pupil area and postoperative pupillary shift under photopic illuminations were measured with the Konan Specular Microscope NSP-9900 (medtec GmbH, Mömbris, Germany). To analyze the amount of reduction of the pupil area and the centration of the iris prosthesis postoperatively, digital images of the eye were taken using the HEYEX (Heidelberg Eye Explorer, Heidelberg Engineering GmbH, Germany). The area of pupillary aperture was calculated in pixels and converted into square millimeters for analysis. The maximum distance between the pupil center and the corneal center was considered as the pupillary shift.

For the evaluation of patient impairment, patients were asked to rate their glare sensitivity on a visual analog scale ranging from 1 to 10 (1 = none, 10 = maximum disturbance) before and after iris reconstruction.

### Data analysis

We used Analyse-it® for Microsoft® Excel 5.11 for all statistical analyses. Decimal visual acuities and contrast sensitivity scores were converted into logMAR and logarithmic units, respectively. Hence, adjusted meter visions such as counting fingers (CF), hand motion (HM), and light perception (LP) were defined as 1.9 (CF), 2.0 (HM) and 2.1 (LP) logMAR.

The non-parametric Wilcoxon test for paired samples was used to compare CDVA, IOP, CS, SE, ECD, pupil area, and patient impairment results. All tests were two-tailed, and significance level was set at a *p*-value of 0.05.

## Results

**Fig. 2:**
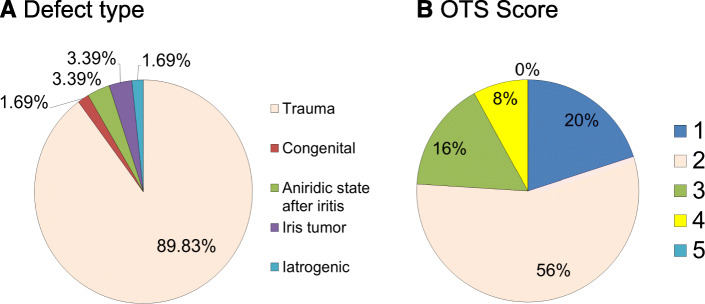
**a** Percentage of defect types (*n* = 59). **b** OTS scores from 1 to 5 (the higher the score, the less trauma; eyes without previous trauma were excluded; *n* = 50) underline the fact that many of the treated eyes were severely pre-damaged

In total, 59 eyes of 56 patients were evaluated in this retrospective observational study, comprising 43 male (76.79%) and 13 female subjects (23.21%). Median age was 56.5 years (range, 28.0 to 84.0 years). Figure 2 shows the percentage of preoperatively recorded types and severities of trauma, respectively. Median CDVA prior to surgery was 0.70 logMAR (range 0.00 to 1.98 logMAR). Median period of time between the diagnosis and the surgery was 37.0 months (range, 1.0 to 79.2 months). Prior to the implantation, 56 iris prostheses were downsized to a smaller diameter (median, 11.50 mm; range, 9.0 to 12.50 mm).

Monocular CDVA at 5 months was significantly better than preoperatively (*p* < 0.0001). 54% (32/59) of patients had an improvement of 0.2 logMAR or greater, 34% (20/59) had an improvement of less than 0.2 logMAR and 12% (7/59) lost 0.2 logMAR or more. 75% of eyes achieved a CDVA better than or equal to 0.7 logMAR. There were no significant changes in IOP values (*p* = 0.881). Photopic and mesopic contrast sensitivity could only be recorded in 36 and 28 eyes, respectively. Logarithmic contrast sensitivity scores under photopic conditions with glare improved significantly from preoperative to postoperative measurements (*p* = 0.008). Under mesopic light conditions with glare, no significant differences were found (*p* = 0.844). Table 1 summarizes the preoperative and postoperative findings.

**Table 1:** IOP, CDVA, contrast sensitivity levels (log) and sensitivity to glare

	Preoperatively	Postoperatively	***p***-value
IOP (mmHg)			
Median (range)	15.0 (8.0 to 30.0)	15.0 (9.0 to 27.0)	0.881
Mean ± SD	15.2 ± 4.0	15.0 ± 3.8	
CDVA (logMAR)			
Median (range)	0.70 (0.0 to 1.98)	0.30 (−0.08 to 2.00)	< 0.0001
Mean ± SD	0.84 ± 0.61	0.55 ± 0.61	
Contrast sensitivity (Pelli-Robson chart)			
Median (range)	0.90 (0.00 to 1.95)	1.35 (0.00 to 1.80)	0.008
Mean ± SD	0.96 ± 0.55	1.26 ± 0.40	
Nyktometer 500			
Median (range)	0.00 (0.0 to 23.5)	0.00 (0.00 to 23.5)	0.844
Mean ± SD	2.07 ± 6.15	2.11 ± 6.12	

### Pupil area and endothelial cell density

Median pupil area was significantly reduced from 51.27 mm^2^ (17.91 to 98.23) to 8.81 mm^2^ (4.16 to 8.84) postoperatively (*p* < 0.0001), representing a median reduction of 79.30%. There was a median pupillary shift of the iris prostheses amounting to 0.28 mm (0.02 to 1.04) from the center of the cornea.

Median ECD also decreased slightly but significantly (*p* = 0.007) from 2646.0 mm^2^ (737.0 to 3145.0) to 2497.5 mm^2^ (728.0 to 3067.0).

### Adverse events and complication management

No adverse events occurred during the surgery procedure. Postoperatively, six eyes (10%) presented complications due to device dislocation (one needed re-fixation by sutures) in four cases and development of glaucoma in two cases which was controlled with conservative medication. Secondary surgery was immediately performed to suture the subluxated iris prostheses resulting in stable and safe positioning. In addition, one eye developed chronic irritation paired with persistent macular edema and corneal decompensation; hence, the artificial iris was explanted after 16 months.

### Questionnaire

Patients were asked to rate subjectively their complaints before and after surgery. Glare was rated from 1 (no glare) to 10 (extreme glare). Preoperatively, median glare score was 9.0 (range 1 to 10; mean 7.9 ± 2.9) and was significantly reduced after surgery to a median score of 4.0 (range 1 to 10; mean 3.9 ± 2.4) (*p* < 0.0001).

## Discussion

If iris prostheses are needed, the eyes are oftentimes severely pre-damaged. Eye injuries in association with iris trauma usually affect other parts of the eye, especially the cornea. This can potentially lead to a loss of vision. In retrospective analyses of patients with major trauma or blunt injuries, study results [2, 12] revealed that the incidence of iris deficiency was 0.5% up to 20.0% including iris sphincter tears and iridodialysis. Insertion of iris prostheses in traumatic eyes should be performed in such a way that an additional trauma is reduced to a minimum (e.g. sclerocorneal incision in eyes with compromised corneas of small corneal incisions in eyes with weak scleral tissue). Without the need of placing fixation sutures, the foldable CUSTOM*FLEX* ARTIFICIAL*IRIS* can be implanted in the posterior chamber using an IOL injector [13].

In our study comprising 59 eyes, penetrating injuries and blunt trauma were the most common mode of injury representing 89.8% of the eyes. Some subjects had undergone preceding surgeries such as pars plana vitrectomy, which sometimes had taken place several years before implantation of the artificial iris.

**Fig. 3:**
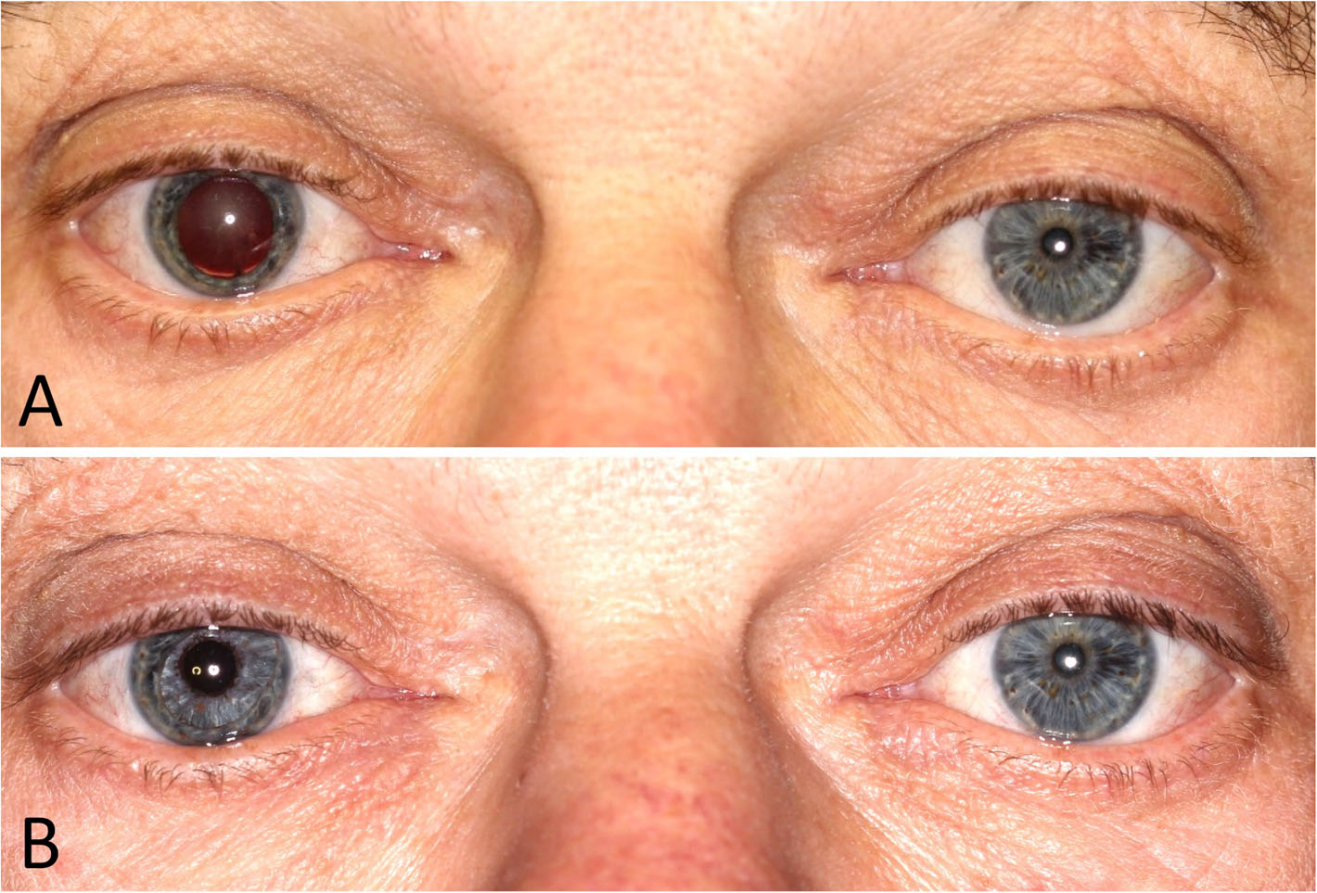
Patient before and after combined artificial iris and IOL implantation (right eye; same patient as in Fig. 1)

Due to the surgical procedure, the median visual acuity initially decreased 2 days after surgery, and then continuously rose at later follow-up visits; however, the initial decrease did not show any significance compared to preoperative measurements. The postoperative visual acuity however, improved significantly. At the follow-up, visual acuity improved in 41 eyes (73%) and remained stable in 2 eyes (4%) but worsened in 13 eyes (23%). Factors for limited increase of postoperative visual outcomes were central corneal scarring and clouding, dislocation of the ArtificialIris, and persistent macular edema. Regarding corneal opacities, Ayliffe et al. [13] claimed that efforts should be made to place the prosthetic pupil in alignment with the clearest, centrally located area of the cornea. However, this could lead to aesthetic dissatisfaction on closer eye inspection. In our study though, patients were subjectively rather affected by glare. More precisely, it primarily seems to be more important to abate the patients’ sensitivity to glare using an appropriate artificial aperture (Fig. 3). However, we also showed in a recent study that aniridia treatment using a custom-made AI prosthesis offers a good aesthetic outcome [14]. Pre- and postoperative photographs of 66 patients were analyzed subjectively and objectively. Patients, eye doctors and laymen rated the overall aesthetic outcome with 8.9 ± 1.4, 7.7 ± 1.1 and 7.3 ± 1.1 out of 10 points, respectively [14].

As already reported in detail [15–17], haptic fixation of a foldable IOL on the back of the ArtificialIris provides advantages regarding sutureless and knotless transscleral fixation. Although remaining foldable, the scleral-fixated IOL-iris complex cannot be inserted using an injector. Nonetheless, the ArtificialIris generally offers the possibility to simultaneously correct aphakia or cataract in aniridia when required, which substantially improves vision. A previous laboratory assessment also showed that suturing of the IOL to the AI can be done in a reliable and reproducible manner without deteriorating optical quality [18].

Compared to normal controls, visual performance of injured eyes appears to be worse in terms of low contrast conditions [19]. Since glare effects and pupil size deviations are crucial factors for the reduction of contrast sensitivity [20], the results in our patients were below the age-related standard contrast sensitivity values in normal persons [21, 22] because of significant traumatic iris tissue loss. In general, postoperative contrast sensitivity improved at photopic and mesopic luminance levels, although this tendency was not statistically significant in mesopic conditions but in photopic. As the overall pupil size of the ArtificialIris amounts to 3.35 mm providing improved contrast sensitivity levels at daytime light conditions, it had to be clarified whether the fixed pupil diameter might be less well adapted to mesopic luminance levels. The results obtained, however, suggest that the predefined size of the pupil does not negatively affect mesopic vision under glare influence.

It is generally known that severe complications after introducing prosthetic iris devices can be as follows: secondary glaucoma, persistent inflammation, retinal detachment, and corneal problems [23–27]. Therefore, proper placement and fixation of the implant is required to avoid mechanical contact between the ArtificialIris and either the lens or the corneal endothelium. According to Gerding et al. [23], insufficient stabilization of the iris prosthesis can cause secondary glaucoma and complete endothelial cell loss with the consequence of penetrating keratoplasty. As expected, low median ECD amounting to less than 2000 cells/mm^2^ was found in 11 eyes before surgical invasion, which fell by an average of 8.41% after implantation of the ArtificialIris. Compared with results in healthy eyes [28], mean endothelial cell loss was within the normal range, although we found a sharp decrease of more than 30% in three eyes. The variable and sometimes great loss of endothelial cells is most likely caused by the time-demanding and rather invasive procedure required. To protect the corneal endothelium in these oftentimes severely pre-damaged eyes, we recommend the extensive use of ophthalmic viscosurgical devices (OVD). As mentioned earlier, when suturing of the iris prostheses was not indicated we used a 2.8 mm incision. We used an approx. 7.0 mm wide sclerocorneal incision to implant the devices when suturing was indicated. We did not notice any differences in rate of complications due to the larger incision, however, we agree with the literature that large incisions should be avoided in eyes with previous trauma [13]. This is particularly necessary to minimize unnecessary additional corneal trauma. In our study cohort, one eye developed secondary corneal decompensation followed by explantation of the ArtificialIris.

After repair of the initial ocular trauma and before ArtificialIris implantation, some of the patients wore commercially available iris print contact lenses to conceal their iris deficiencies, but with partly limited success due to translucent printing, incompatibility reaction, and poor compliance. Despite having intraocular intervention, we therefore assume that the ArtificialIris offers better results regarding handling, cosmetic [9] and functional rehabilitation on a continuing basis and reduces risk for contact lens induced complications to the cornea. Moreover, this prosthetic iris device has proven to be convenient in ocular comorbidities [5, 8, 13].

## Conclusion

To sum up, the implantation of an ArtificialIris in combination with an individual IOL can be a life-enhancing procedure. Glare disability is significantly decreased, severe photophobia is eliminated and refractive disorders are corrected. The described approach offers an additional option to surgeons to treat partial or total aniridia as well as aphakia in one single surgery. Finally, our observational case series confirmed the findings of previous reports showing very few severe adverse events regarding ArtificialIris implantation, if cautious handling and application is warranted.

## Data Availability

The data used to support the findings of this study are available from the corresponding author upon request.

## Abbreviations

CDVA: Corrected distance visual acuity
CE: European certification mark
CF: Counting fingers
CS: Contrast sensitivity
ECD: Endothelial cell density
FDA: U.S. Food and Drug Administration
HM: Hand motion
IOL: Intraocular lens
IOP: Intraocular pressure
logMAR: logarithm of the Minimum Angle of Resolution
LP: Light perception
OTS: Ocular trauma score
PMMA: Poly (methyl-methacrylate)
